# Effects of Female Community Health Volunteer Capacity Building and Text Messaging Intervention on Gestational Weight Gain and Hemoglobin Change Among Pregnant Women in Southern Nepal: A Cluster Randomized Controlled Trial

**DOI:** 10.3389/fpubh.2020.00312

**Published:** 2020-07-17

**Authors:** Jitendra Kumar Singh, Dilaram Acharya, Rajan Paudel, Salila Gautam, Mandira Adhikari, Shambhu Prasad Kushwaha, Ji-Hyuk Park, Seok-Ju Yoo, Kwan Lee

**Affiliations:** ^1^Department of Community Medicine, Janaki Medical College, Tribhuvan University, Janakpur, Nepal; ^2^Department of Preventive Medicine, College of Medicine, Dongguk University, Gyeongju-si, South Korea; ^3^Department of Community Medicine, Devdaha Medical College and Research Institute, Kathmandu University, Dhulikhel, Nepal; ^4^Central Department of Public Health, Institute of Medicine, Tribhuvan University, Kathmandu, Nepal; ^5^Department of Public Health, Sanjeevani College of Medical Sciences, Biratnagar, Nepal; ^6^Nepal Development Society, Bharatpur, Nepal; ^7^District Health Office, Janakpur, Nepal

**Keywords:** capacity building, community health volunteers, gestational weight gain, hemoglobin changes, text messaging

## Abstract

**Introduction:** Public health interventions such as text messaging are commonly evaluated in high-income countries and that the evaluation reports of the effectiveness of community health volunteers in low-income countries like Nepal is scarce. This study aimed to determine whether female community health volunteer (FCHV) capacity building and text messaging to expectant mother increases gestational weights and hemoglobin levels of pregnant women living in southern Nepal.

**Methods:** A cluster randomized control trial was carried out in 52 clusters of 6 Village Development Committees in southern Nepal between July 2015 and March 2016. A total of 413 pregnant mothers of gestation age between 13 and 28 weeks (214 in the intervention group and 199 in the control group) were included in the analysis. Intervention consisted of FCHV capacity building followed by regular supervision and monitoring and mobile phone text messaging to expectant mothers. Regression analysis, controlled for confounders, was conducted to assess gestational weight gains and changes in hemoglobin levels.

**Results:** At the end of the pregnancy, the mean weight gain difference between the intervention and control groups was 1.1 kg (95% CI: 1.0, 1.9). Rates of weight increases in the intervention and control groups were 0.504 kg/week (95% CI: 0.371, 0.528), and 0.399 kg/week (95% CI: 0.362, 0.465), respectively. Similarly, the mean inter group difference in hemoglobin levels was 0.11 gm/dl (95% CI: 0.09, 0.15), and rates of hemoglobin increases (gm/dl/week) in the intervention and control groups were 0.02 gm/dl (95% CI: 0.01, 0.09) and 0.004 gm/dl (95% CI: 0.02, 0.12), respectively.

**Conclusions:** The study shows that FCHV capacity building and mobile text messaging have a positive effect on the gestational weights and hemoglobin levels of expectant mothers. Our findings suggest that mobile text messaging coupled with FCHV capacity building services should be supported and would usefully expand in resource poor settings.

**Trial registration:** ISRCTN60684155.

## Introduction

Despite remarkable progress worldwide in maternal and child health (MCH) over the past decade, mortality due to maternal-associated causes stands at one per 180 women, and in 2015, the under-five mortality rate (U5MR) was reported to be 43 per 1,000 live births and the neonatal mortality rate (NMR) to be 19 per 1,000 live births (1). The major leading reported causes of maternal deaths are obstetric (e.g., hemorrhage, hypertensive disorder during pregnancy, and sepsis) or non-obstetric (e.g., anemia and HIV/AIDs). On the other hand, prematurity, intra partum-related complications, and sepsis are three most common causes of increased neonatal deaths (2). Studies have consistently reported that poor maternal weight gain and low maternal hemoglobin level during pregnancy are significantly associated with adverse maternal health outcomes such as increased risk of preterm premature rupture of membranes, gestational cholestasis, postpartum hemorrhage, preeclampsia, the need for blood transfusion) (3, 4) and poor child health outcomes (low birth weight (LBW), preterm birth, small-for-gestational-age (SGA), stillbirth, and perinatal and neonatal mortality) (3–5). However, fortunately, the majority of maternal and child morbidities and mortalities can be prevented by raising maternal education and income, decreasing birth rates, and improving nutritional status (6–8).

Prior studies being conducted in developing countries demonstrated that the capacity enhanced community health volunteers can contribute to improve maternal and child health (MCH) status (9–11), while other studies from both of developing and developed countries reported the effectiveness of text messaging interventions to increase health service utilization rates and MCH status (12–16). For instance, Khorshid et. al. (15) reported text messaging resulted higher iron supplement compliance among pregnant women, while others (12–14) have reported the use of text messaging to educate people about healthy living practices had beneficial impacts on disease prevention and control and on health service utilization rates. Studies conducted in Tanzania also showed that ANC visit rates and the rate of utilization of skilled health workers during delivery was increased by the utilization of text messaging services (17, 18). Furthermore, Avery et. al. concluded that enhancing the capacity and effectiveness of community health volunteers positively impacted the utilization of maternal and child care services in Kenya (10).

More than 50,000 Female Community Health Volunteers (FCHVs) in Nepal work on health promotion and disease prevention, and many health interventions implemented by these volunteers have improved maternal and child health outcomes (19). The Ministry of Health and Population (MOHP) Nepal has launched several maternal health interventions such as antenatal check-ups, iron and folic acid supplementation, and de-worming tablets, and provided nutritional counseling to expectant mothers through existing public health facilities (20). However, the prevalence of anemia among pregnant women remains high and declined only minimally between 2011 and 2016 (from 48 to 46%), and LBW rates remained stagnant over the same period at 12% (21). Such statistics show that extensive efforts are needed in Nepal to address the issues of LBW and maternal anemia.

Mobile phone use in Nepal has increased exponentially over the last decade to 113% per capita in 2017 from 21% in 2009 (22), and trained FCHVs have successfully utilized this device for health promotion and disease prevention (19). Given this background, we aimed to determine whether female community health volunteer capacity building and text messaging to expectant mothers might be used to increase gestational weights and hemoglobin levels among pregnant women in southern Nepal.

## Materials and Methods

### Study Setting

This cluster randomized controlled trial (CRCT) was conducted in Dhanusha district of southern Nepal between July 2015 and March 2016. At baseline, 426 pregnant women in their second trimester (gestation period 13–28 weeks) aged 15–45 years residing in 52 clusters (wards) in six Village Development Committees (VDCs) of Dhanusha district of Nepal were included. Dhanusha is one of 77 districts in Nepal and is situated in the Southern Terai region. VDCs are administrative units below the district level and are further divided into smaller units called wards (23). Each ward functioned as a cluster in the present study. The details of methods used for CRCT were previously described elsewhere (24).

### Sampling

The sample size for this “MATRI-SUMAN” trial (24) was estimated using the formula for a two parallel arms cluster randomized controlled trial with a power of 80% and a level of significance of 5% considering the previous study on skilled birth attendant (SBA). The rate of SBA utilization was 12% in the Dhanusha district, and a minimum intergroup difference on SBA utilization was of 20% after intervention (25). Considering a design effect of 2.29 to account for intra-cluster correlation and a possible dropout rate of 20%, an adjusted sample size of 354 participants was computed. However, in order to maximize the sample size for the interventional study, 426 eligible pregnant women were included at baseline. Participants were selected using the multi-stage sampling technique. Initially, we selected two primary health care facilities (one Health Post and one Primary Health Care Centre) at which there was no externally funded international or national non-governmental organization representatives working on improving maternal and child outcomes.

Health Post is the smallest unit and first contact with health care delivery system of Nepal for basic health services; each level above the health post level is a referral point in a network from PHCCs on to the primary and secondary level hospitals, and finally to tertiary level hospitals (26). Second, 52 wards (clusters) from six VDCs representing a population of 66,000 were selected from the catchment areas of these two health care facilities (23, 27). Finally, every pregnant women(gestation period between 13 and 28 weeks) in the selected 52 clusters were included in this trial with the help of FCHVs (28). At baseline, 426 pregnant women from these 52 clusters were eligible for the study.

Of 426 pregnant women recruited at baseline 219 were allocated to the intervention group and 207 to the control group. Clusters were randomized to the intervention and control groups by simple assignment using a random number sequence. Neither researchers nor participants were blinded due to the nature of the study.

**Figure 1:**
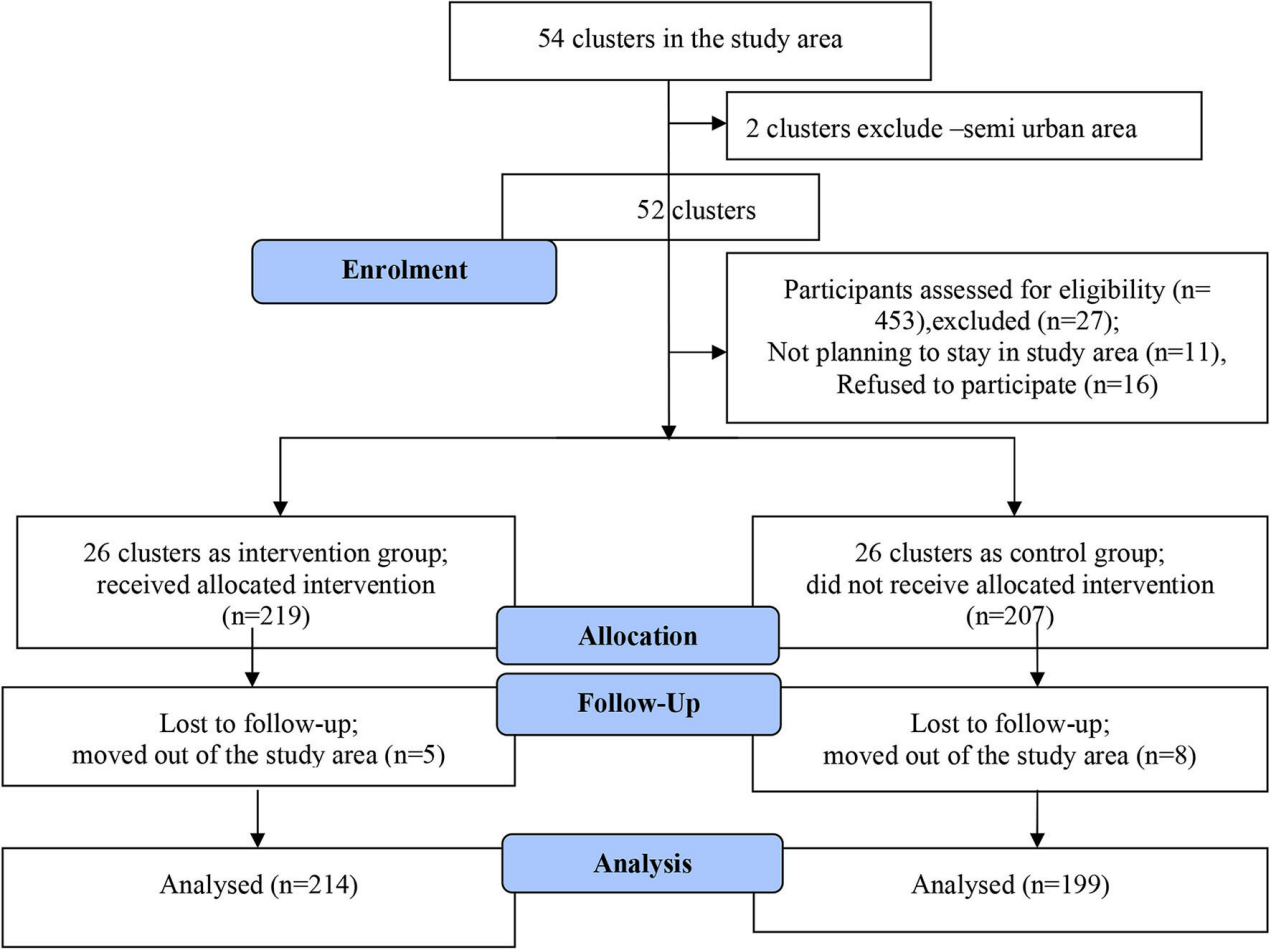
Study CONSORT flow diagram.

Altogether, 13 participants were lost to follow up (nine participants moved out of the study area and four women had miscarriages), the remaining 413 participants (214 in the intervention group and 199 in the control group) constituted the study cohort and were included in the analysis (Figure 1). The adequacy of sample size for investigating the key components of MCH care (gestational weight gain and hemoglobin changes) was assessed using *post-hoc* power calculations using an online application (29). Power calculations were made by comparing means of outcome variables (gestational weight gain and hemoglobin changes) in the intervention and control groups.

### Interventions

**Figure 2:**
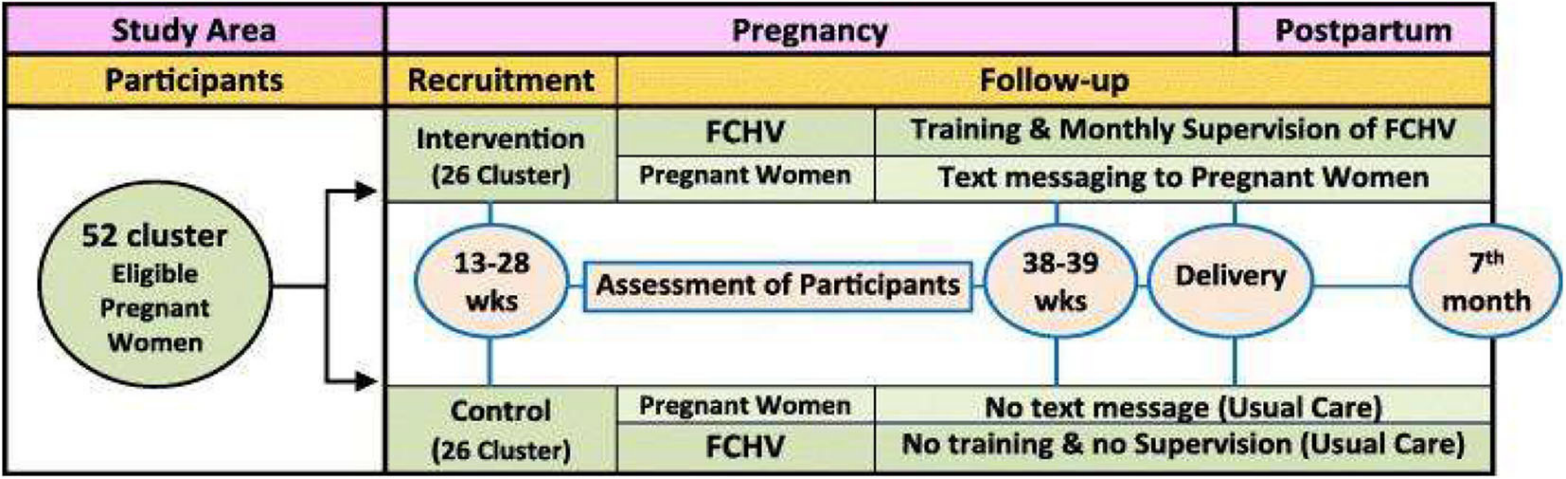
Schematic of the intervention study design.

Intervention was performed using two approaches, that is, by FCHV capacity building followed by regular supervision and monitoring and by text messaging via mobile phone. FCHV capacity building was performed by providing 1 day of reinforcement training. Training documents included materials regarding the MCH services to be utilized and recommended maternal diets. Monthly supervision and monitoring of FCHVs were also performed to assess performances. Text messages were sent to pregnant women regarding MCH service utilization and dietary intake during pregnancy and postpartum. Text messages were sent in Nepali (the local language) during evenings or mornings according to participant choice. Messages were sent at a rate of one message per fortnight between the 4th and 6th months and weekly thereafter till childbirth (Figure 2). Themes for text messages and capacity building training were adapted from the documents: The birth preparedness packages used were “Jeevan Suraksha” developed by United States Agency for International Development(USAID) (Nepal), “The Micronutrient Initiative” Nepal, and Bal Pariwar Mitra (India) (30, 31). Details of interventions were as previously described (24).

### Measurements and Data Collection

#### Weight Gain and Hemoglobin Concentration Measurements

Weight and serum samples were obtained from all 413 participants at first prenatal visits (13–28 weeks of gestation) and at the end of pregnancy (38–39 weeks). Weights were obtained using calibrated scales to the nearest 0.1 kg with subjects wearing light indoor clothes without footwear. Blood samples obtained from participants were collected in ethylenediamine tetra-acetic acid (EDTA) vials and sent to the clinical laboratory of the Janaki Health Care and Research Center on same days to measure serum hemoglobin levels using the *Cyanmethemoglobin* method (32). Socio-demographic characteristics such as age, caste/ethnicity, religion, education, wealth index, and parity were collected by face-to-face interview using a structured questionnaire as these variables are defined in our previously published paper (28).

### Ethics

Written informed consent (signature from those who were literate and thumb print for illiterate participants) was obtained from all eligible participants or their guardians (in case of minor) after providing them an explanation of study objectives and procedures. Ethical approvals were obtained from the Ethical Review board of the Nepal Health Research Council (approval number: 101), the Ethics Committee of Banaras Hindu University, Varanasi, India (approval number: ECR/526/Inst/UP/2014 Dt.31.1.14), and from the District Public Health Office, Dhanusha, Nepal (approval number: 2245).

### Statistical Analysis

The Chi-square test to investigate homogeneities of categorical baseline characteristics in the intervention and control groups, and the independent *t*-test to determine the significance of weight gain and hemoglobin level (treated as continuous variables) differences between the two study groups. Patterns of weight gains and hemoglobin changes were assessed by using mixed-effects models. Data were entered into Epi Data 3.1 Software, and the analysis was conducted using Statistical Package for Social Sciences version 22.0 (SPSS, IBM, Armonk, NY, USA). Statistical significance was accepted for *p* < 0.05.

## Results

Table 1 presents the personal characteristics of the 413 study participants, of which more than two thirds (69.0%) were aged from 20 to 34 years. Of the 413 participants 61.5% were from an upper caste, 91.8% believed in the Hindu religion, 25.2% were illiterate, and 27.1% were engaged in household work. Most participants (61.1%) were multiparous, and 29.3% were in the lowest wealth quintile (29.3%). Univariate analysis showed, with the exceptions of age and parity, the socio-economic characteristics of the intervention and control groups were similar.

**Table 1:** Personal characteristics of the intervention and control groups.

**Characteristics**	**Total** ***N* = 413 (%)**	**Intervention group** ***n =* 214 (%)**	**Control group** **n = 199 (%)**	***p*-value**
**Age of mothers**				
≤19 years	94 (22.8)	43 (20.1)	51 (25.6)	*P* = 0.045
20–34 years	285 (69.0)	147 (68.7)	138 (69.3)	
≥35 years	34 (8.2)	24 (11.2)	10 (5.0)	
**Caste/ethnicity**				
Dalit	68 (16.5)	35 (16.4)	33 (16.6)	*P* = 0.587
Aadibasi/Janajati^*^	91 (22.0)	43 (20.1)	48 (24.1)	
Upper caste group^**^	254 (61.5)	136 (63.5)	118 (59.3)	
**Religion**				
Hindu	379 (91.8)	198 (92.5)	181 (91.0)	*P* = 0.708
Muslim	25 (6.1)	11 (5.1)	14 (7.0)	
Buddhist/Christian	9 (2.2)	5 (2.4)	4 (2.0)	
**Mother's education**				
No education	104 (25.2)	50 (23.4)	54 (27.1)	*P* = 0.164
Primary	148 (35.8)	83 (38.8)	65 (32.7)	
Secondary	78 (18.9)	45 (21.0)	33 (16.6)	
Higher	83 (20.1)	36 (16.8)	47 (23.6)	
**Mother's occupation**				
No work/household works	112 (27.1)	60 (28.0)	52 (26.1)	*P* = 0.102
Agriculture/farming	130 (31.5)	70 (32.7)	60 (30.2)	
Service/salaried	33 (8.0)	15 (7.0)	18 (9.0)	
Sales/business	59 (14.3)	22 (10.3)	37 (18.6)	
Manual/waged labor	79 (19.1)	47 (22.0)	32 (16.1)	
**Wealth index**				
1st quintile	121 (29.3)	70 (32.7)	51 (25.6)	*P* = 0.57
2nd quintile	99 (24.0)	48 (22.4)	51 (25.6)	
3rd quintile	79 (19.1)	37 (17.3)	42 (21.1)	
4th quintile	59 (14.3)	27 (12.6)	32 (16.1)	
5th quintile	55 (13.3)	32 (15.0)	23 (11.6)	
**Parity**				
Primiparous	161 (39.0)	70 (32.7)	91 (45.7)	*P* = 0.007
Multiparous	252 (61.0)	144 (67.3)	108 (54.3)	

* *Relatively disadvantaged*;

** *relatively advantaged*.

The weights and serum hemoglobin levels of participants in the intervention and control groups at baseline and after intervention are provided in Table 2. Mean [standard deviation (SD)] weights and hemoglobin levels at baseline (13–28 weeks of gestation) in the intervention group were 45.93 kg (5.90 kg) and 11.20 mg/dl (1.08 mg/dl), respectively, and corresponding values in the control group were 45.66 kg (5.52 kg) and 11.0 mg/dl (1.15 mg/dl), respectively. At baseline, mean weight and hemoglobin level (*p* = 0.214) were similar in the two groups (*p* = 0.624 and 0.214, respectively). However, after intervention (at 38–39 weeks of gestation) mean weights and hemoglobin levels in the intervention and control groups were 52.36 kg (6.28 kg) and 50.94 kg (5.90 kg) and 11.43 mg/dl (1.00 mg/dl) and 11.11 mg/dl (1.10 mg/dl), respectively, and these values were significantly different (*p* = 0.018 and 0.024, respectively).

**Table 2:** Body weights and hemoglobin levels of members of the intervention and control groups at baseline and after intervention.

**Variables**	**Total (*n* = 413) Mean (SD)**	**Intervention group (*n* = 214)** **Mean (SD)**	**Control group (*n* = 199)** **Mean (SD)**	***p-*value**
**At baseline (13–28 weeks of gestations)**				
Weight (kg)	45.80 (5.72)	45.93 (5.90)	45.66 (5.52)	*P* = 0.624
Hemoglobin level (g/dl)	11.12 (1.07)	11.20 (1.08)	11.0 (1.15)	*P* = 0.214
**After intervention (38–39 weeks of gestation)**	**Mean (SD)**	**Mean (SD)**	**Mean (SD)**	
Weight (kg)	51.68 (6.13)	52.36 (6.28)	50.94 (5.90)	*P* = 0.018
Hemoglobin level (g/dl)	11.28 (1.01)	11.43 (1.00)	11.11(1.10)	*P* = 0.024

*SD, standard deviation; kg, Kilogram; g/dl, gram/decilitre; n, numbers; baseline hemoglobin level (gm/dl), intervention hemoglobin level (gm/dl)*.

Multivariable analyses of gestational weight gains and hemoglobin changes are provided in Tables 3, 4. After adjusting for age, caste, religion, education, occupation, level of wealth, parity, and body mass index, mean weight gains in the intervention and control groups were 6.9 kg (95% CI: 6.2, 7.7) and 5.8 kg (95% CI: 5.2, 5.9), respectively; a mean difference of 1.1 kg (95% CI: 1.0, 1.9). Rates of change of weight in intervention and control groups were 0.504 kg/week (95% CI: 0.371, 0.528) and 0.399 kg/week (95% CI: 0.362, 0.465), respectively (Table 3).

**Table 3:** Gestational weight gains in the intervention and control groups.

**Weight gain (kg)**	Intervention group (*n =* 214/428^*^)	Control group (*n =* 199/398^*^)	***p*-value**
**Unadjusted (COR)**			
Total weight gain, kg (95% CI)	6.4 (6.1, 6.7)	5.2 (5.0, 5.5)	<0.0001
Mean difference, kg (95% CI)	1.2 (1.0, 1.6)	Reference	
Rates of weight gain, kg/week (95% CI)	0.42 (0.32, 0.47)	0.32 (0.31,0.43)	<0.0001
Adjusted (aOR)^**^			
Total weight gain, kg (95% CI)	6.9 (6.2, 7.7)	5.8 (5.2, 5.9)	<0.0001
Mean difference, kg (95% CI)	1.1 (1.0, 1.9)	Reference	
Rates of weight gain, kg/week (95% CI)	0.50 (0.37, 0.52)	0.39 (0.36, 0.46)	<0.0001

*CI, confidence interval; Kg, kilogram*;

* n represents the number of participants/measurements; COR, crude odds ratio; aOR, adjusted odds ratio

** *adjusted for age, caste/ethnicity, religion, education, occupation, wealth quintile, and parity*.

**Table 4:** Changes in serum hemoglobin levels in intervention and control groups.

**Hemoglobin level (gm/dl)**	Intervention group (*n =* 112/224^*^)	**Control group** (*n =* 100/200^*^)	***p*-value**
**Unadjusted (COR)**			
Total Hb change, gm/dl (95% CI)	0.23 (0.17,0.29)	0.11 (0.2, 0.18)	0.016
Mean difference, gm/dl (95% CI)	0.12 (0.08, 0.21)	Reference	
Rates of Hb change, gm/dl/week (95% CI)	0.015 (0.011, 0.07)	0.006 (0.003, 0.009)	<0.0001
Adjusted (aOR)^**^			
Total Hb change, gm/dl (95% CI)	0.21 (0.13,0.22)	0.10 (0.07, 0.13)	<0.0001
Mean difference, gm/dl (95% CI)	0.11 (0.09, 0.15)	Reference	
Rates of Hb change, gm/dl/week (95% CI)	0.02 (0.01, 0.09)	0.004 (0.002, 0.12)	<0.0001

*CI, Confidence interval; Hb, hemoglobin; gm, gram*;

* n represents the number of participants/measurements; COR, crude odds ratio; aOR, adjusted odds ratio

** *adjusted for age, caste/ethnicity, religion, education, occupation, wealth quintile, and parity*.

Mean total serum hemoglobin changes in the intervention and control groups were 0.21 gm/dl (95% CI: 0.13, 0.22) and 0.10 gm/dl (95% CI: 0.07, 0.13); a mean difference of 0.11 gm/dl (95% CI: 0.09, 0.15), and corresponding rates of change of hemoglobin were 0.02 gm/dl (95% CI: 0.01, 0.09), and 0.004 gm/dl (95% CI: 0.002, 0.12), respectively (Table 4).

### Subgroup Analysis

We did subgroup analysis for changes in weight and serum hemoglobin levels at baseline and after intervention in intervention and control group by caste/ethnicity and educational status, demonstrated in Tables 5, 6.

**Table 5:** Changes in weight and serum hemoglobin levels in intervention and control group at baseline and after intervention by caste/ethnicity.

**Caste/ethnicity**		**Group**	**Weight (kg)**			**Hemoglobin (g/dl)**		
			***N***	**Mean (SD)**	***p-*value**	***N***	**Mean (SD)**	***p*-value**
Upper caste group	At baseline	Intervention	136	45.85 (5.30)	0.218	82	11.25 (1.01)	0.288
		Control	118	46.65 (5.06)		63	11.07 (1.08)	
	After intervention	Intervention	136	52.13 (5.56)	0.821	70	11.50 (0.99)	0.023
		Control	118	51.97 (5.57)		54	11.09 (0.93)	
Aadibasi/Janajati	At baseline	Intervention	43	46.98 (7.96)	0.104	26	11.08 (0.90)	0.605
		Control	48	44.60 (5.74)		26	11.22 (1.16)	
	After intervention	Intervention	43	53.72 (8.06)	0.018	21	11.22 (0.93)	0.586
		Control	48	50.17 (5.97)		22	11.37 (0.94)	
Dalit	At baseline	Intervention	35	45.00 (5.09)	0.320	25	11.12 (1.15)	0.412
		Control	33	43.64 (6.11)		26	10.81 (1.29)	
	After intervention	Intervention	35	51.63 (6.39)	0.038	21	11.42 (1.14)	0.184
		Control	33	48.39 (6.16)		24	10.92 (1.19)	

*N, number; SD, standard deviation; kg, Kilogram; g/dl, gram/decilitre*.

**Table 6:** Changes in weight and serum hemoglobin levels in intervention and control group at baseline and after intervention by educational status.

**Education**		**Group**	**Weight (kg)**			**Hemoglobin (g/dl)**		
			***N***	**Mean (SD)**	***p*-value**	***N***	**Mean (SD)**	***p*-value**
No education	At baseline	Intervention	50	45.46 (6.07)	0.528	31	11.21 (1.11)	0.222
		Control	54	44.65 (6.93)		33	10.85 (1.25)	
	After intervention	Intervention	50	52.68 (6.60)	0.020	23	11.44 (1.24)	0.179
		Control	54	49.46 (7.18)		31	10.99 (1.17)	
Primary	At baseline	Intervention	83	46.84 (6.60)	0.124	54	11.20 (1.03)	0.691
		Control	65	45.37 (4.44)		31	11.10 (1.15)	
	After intervention	Intervention	83	52.78 (6.85)	0.043	49	11.42 (0.97)	0.257
		Control	65	50.74 (4.81)		25	11.15 (0.92)	
Secondary	At baseline	Intervention	45	45.13 (5.69)	0.609	25	10.82 (0.81)	0.429
		Control	33	45.70 (3.99)		16	11.05 (1.06)	
	After intervention	Intervention	45	50.60 (6.18)	0.760	20	10.96 (0.64)	0.294
		Control	33	50.97 (4.46)		13	11.21 (0.70)	
Higher	At baseline	Intervention	36	45.50 (3.78)	0.134	23	11.57 (0.87)	0.160
		Control	47	47.19 (5.81)		35	11.18 (1.09)	
	After intervention	Intervention	36	53.17 (3.97)	0.818	20	11.89 (0.90)	0.012
		Control	47	52.89 (6.16)		31	11.16 (1.02)	

*SD, standard deviation; kg, Kilogram; g/dl, gram/decilitre*.

After intervention, a significant difference was observed for mean (SD) weight among dalit and adibasi/janajati ethnicity [51.63 kg (6.39 kg) vs. 48.39 kg (6.16 kg) and 53.72 kg (8.06 kg) vs. 50.17 kg (5.97 kg) in intervention and control group, respectively; (*p* < 0.05)]. On the other hand, for mean (SD) hemoglobin level significant difference was observed among upper caste group [11.50 mg/dl (0.99 mg/dl) vs. 11.09 mg/dl (0.93 mg/dl) in intervention and control group, respectively; *p* = 0.023] (Table 5).

After intervention, a significant difference was observed for mean (SD) weight among pregnant women who were illiterate and primary education [52.68 kg (6.60 kg) vs. 49.46 kg (7.18 kg) and 52.78 kg (6.85 kg) vs. 50.74 kg (4.81 kg) in intervention and control group, respectively; (*p* < 0.05)]. On the other hand, hemoglobin level significantly differs among pregnant women having higher level of education [mean (SD): 11.89 mg/dl (0.90 mg/dl) vs. 11.16 mg/dl (1.02 mg/dl) in intervention and control group, respectively; *p* = 0.012] (Table 6).

## Discussion

In this community-based cluster randomized controlled trial, we found that capacity building of female community health volunteers coupled with mobile text messaging to pregnant women significantly increased gestational weights and serum hemoglobin levels as compared to matched controls.

The study shows that nutritional statuses, as reflected by body weight and serum hemoglobin, of expectant mothers are likely to improve when they are continually supervised by trained FCHVs that forward nutrition-related mobile text messages like “you should consume IFA tablets daily from the 4th month of pregnancy until 45 days after delivery (total 225 tablets), take one dose of de-worming tablets during the 4th month of pregnancy, eat diverse foods (cereals, pulses, vegetables, green leafy vegetables, fruit, milk and milk products, and egg/fish/meat or sprouted legumes), and take adequate rest and sleep and avoid hard work.” In addition, members of the intervention group also received periodic mobile text messages regarding the recommended level of antenatal care visits to ensure they received adequate natal and post-natal services.

The positive effect of intervention on gestational weight gains and hemoglobin levels was probably due to greater awareness of health-related issues. Previous studies (12–14, 17, 18) have demonstrated mobile text messaging offers an excellent means of educating people about healthy living practices and healthy behaviors and of increasing MCH service utilization rates. A recent systemic review (33) conducted in low and middle income countries also supported the notion that text or voice messaging reminders improve antenatal care attendance, postnatal care attendance, and childhood immunization rates. In fact, several studies have concluded that mobile text messaging intervention helps maintain appropriate gestational weight and compliance with iron supplements among pregnant women and improve their utilization of MCH care services, especially in rural settings (15, 34, 35). Another study (36) on the effectiveness and acceptance of repeated texted nutritional messages in college students showed increased knowledge of nutrition and of recommended fruit and vegetable consumption and that students were amenable to receiving such messages.

Similarly, capacity building of community health volunteers has been found to have equally effective for the increased uptake of maternal and child health care services. A Kenyan study demonstrated that capacity enhancement of community health volunteers using simple and scalable Monitoring and Tracking Tool (MMATT) and training improved maternal, new-born and child health (MNCH) outcomes (10). Likewise, Tanzanian study also reported that Village Health Workers' capacity-building and empowerment in regard to the MCH services was found to be effective to improving maternal health indicators (37). More interestingly, two recent qualitative studies from Nepal reported that the better contribution of FCHVs in MCH care when they are provided regular training and have access to medical supplies, and also advocated the communication training need be in place to all FCHVs and local health care providers in order to reach to the minority groups (11, 38).

Our study also tested the effectiveness of the intervention with reference to some of the important participants' personal attributes such as caste/ethnicity and level of education by having sub-group analysis (Tables 5, 6). In subgroup analyses, we observed the increase in weight gain among study subjects who were from dalit and adibasi/janajati group, and illiterate and primary level education holders in intervention group. On the other hand, there was increase in hemoglobin level among those expectant mothers who were from upper caste group and having higher education level in intervention group. However, in our final multivariate model, we adjusted such important significant factors to observe the difference between intervention and control group for weight gain and hemoglobin changes.

However, mobile text messaging interventions are not without problems. Although many papers, including systematic reviews, have reported that mobile health interventions including text messaging are efficacious enough in terms of reducing physical and mental health problems such as substance and drug abuse and maternal and child health problems (39–42), power supply deficits and poor mobile network connectivity, which are commonplace in developing countries often prevent access to such interventions (43).

The present study has some specific strengths, that is, it had a robust design, relatively few of the initially recruited participants dropped out. Nonetheless, the study also has its limitations. First, almost one third of our study participants had no education that could have influenced the uptake of the text messaging. Second, the study was performed in one district, which threatens its external validity. We suggest multicentre RCTs be conducted to confirm the effectiveness of mobile text messaging and health volunteer capacity building on maternal and child health outcomes in Nepal.

## Conclusions

The present study demonstrates that FCHV capacity building and mobile text messaging to expectant mothers positively influences gestational weights and maternal serum hemoglobin levels. Our findings suggest mobile text messaging coupled with FCHV capacity building should be supported and that their use be expanded in poor resource settings. Further research is required to determine the cost-effectiveness of FCHV capacity building and mobile text messaging and the willingness of expectant mothers to participate in Nepal.

## Data Availability Statement

The raw data supporting the conclusions of this article will be made available by the authors, without undue reservation.

## Author Contributions

JS, DA, and RP conceptualized the study, performed statistical analysis, and drafted the primary manuscript. SG, MA, SK, J-HP, S-JY, and KL contributed with the significant inputs for data analysis and interpretation of results, and subsequent revision of the contents of the manuscript. Finally, all authors read and approved the final version of the manuscript.

## Conflict of Interest

The authors declare that the research was conducted in the absence of any commercial or financial relationships that could be construed as a potential conflict of interest.
